# Diffused Matrix Format: A New Storage and Processing Format for Airborne Hyperspectral Sensor Images

**DOI:** 10.3390/s100504996

**Published:** 2010-05-18

**Authors:** Pablo Martínez, Alejandro Cristo, Magaly Koch, Rosa Mª. Pérez, Thomas Schmid, Luz M. Hernández

**Affiliations:** 1 Escuela Politécnica de Cáceres, Universidad de Extremadura, Avda. Universidad s/n, 10071, Cáceres, Spain; E-Mails: acristo@unex.es (A.C.); rosapere@unex.es (R.P.); luzhder@unex.es (L.H.); 2 Center for Remote Sensing, Boston University, 725 Commonwealth Avenue, Boston, MA, USA; E-Mail: mkoch@bu.edu; 3 CIEMAT, Avda. Complutense 22, 28040, Madrid, Spain; E-Mail: thomas.schmid@ciemat.es

**Keywords:** hyperspectral images, linear spectral unmixing, remote sensing data format

## Abstract

At present, hyperspectral images are mainly obtained with airborne sensors that are subject to turbulences while the spectrometer is acquiring the data. Therefore, geometric corrections are required to produce spatially correct images for visual interpretation and change detection analysis. This paper analyzes the data acquisition process of airborne sensors. The main objective is to propose a new data format called Diffused Matrix Format (DMF) adapted to the sensor's characteristics including its spectral and spatial information. The second objective is to compare the accuracy of the quantitative maps derived by using the DMF data structure with those obtained from raster images based on traditional data structures. Results show that DMF processing is more accurate and straightforward than conventional image processing of remotely sensed data with the advantage that the DMF file structure requires less storage space than other data formats. In addition the data processing time does not increase when DMF is used.

## 1. Introduction

Earth observation sensors produce daily an immense amount of information, which is stored in the form of different data file types that require enormous amounts of storage space. Most of these data files are needed for conducting many present-day applications. However, some of the acquired images are not necessarily used immediately and therefore, need to be stored for future uses, especially those concerning change detection studies [1].

The massive storage space required for images obtained by these sensors makes it necessary to develop new standard formats that minimize their storage space by including some pre-processing steps that may significantly simplify later comparison with different images and ground truthing information. One of the main applications that makes ample use of the enormous amount of Earth observation information currently being collected, is the study of changes occurring on the Earth’s surface. However, data fusion is mandatory because the acquisition conditions may not be exactly the same due to variations in weather conditions, instrument calibration, or modifications in the trajectory and height of the platform that carries the sensor.

Digital remote sensing data are stored as a set of files containing the description of the data (metadata), pixel values and spatial information. Current types of available data formats store the data by using matrices that hampers the work with images obtained from different sensors. Therefore, new data formats should facilitate the comparison of images for different acquisition dates, flights and/or orbits. In addition, new data formats should make on-board processing easier [2]. However, on-board processing requires that data structures integrate the spatial information provided by the position hardware with the continuous flow of spectral information supplied by the different instruments carried on the platform [3].

The original data as collected by remote sensing instruments are in Level 0 (L0) format (product levels according to Committee on Earth Observation Satellites–CEOS). They are usually pre-processed before being delivered to the user. Image pre-processing includes several operations such as noise reduction, radiance conversion, and geometric correction [4]. Geometric correction is necessary to generate a map projection of the L0 image by removing any pixel misalignment. The geometry of the acquisition depends strongly on the sensor configuration. Whisk broom sensors (*i.e.,* AVIRIS) have only one detector, which scans the scene by using mirrors. The coordinate system used by this sensor is hemispherical and the size of the sensor's footprint (integration area of the pixel) depends on the distance from the platform to the target. Push broom sensors (*i.e.,* ROSIS) have an array of detectors, which are placed transversally to the platform movement. The coordinate system for this sensor is cylindrical and the size of the sensor's footprint depends on the distance of the cylindrical axis to the target. Image sensors, on the other hand, have a matrix of detectors with a Cartesian coordinate system and the size of the sensor's footprint depends on the coordinates *i* and *j* of the target [5]. All these sensors have circular footprints for the nadir target and ellipsoidal footprints for the off-NADIR positions.

**Figure 1.**
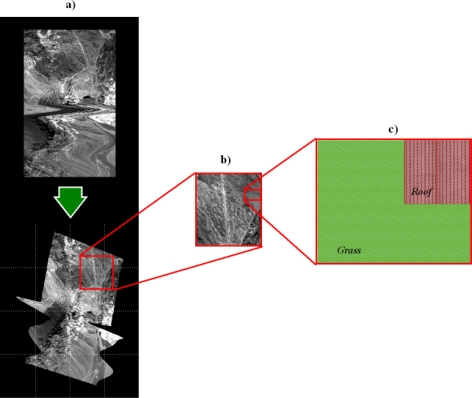
Image acquired by an airborne sensor, shown at different scales.

When acquiring an airborne image it is necessary to take into account that the platform can be subjected to movements, so a Ground Position System (GPS) or Inertial Navigation System (INS) system can be helpful in restoring the data according to the location of the measurements. In this way, a geo-correction process, like the one in Figure 1a, is required. Figure 1b and c are magnified regions of the geo-corrected image used in this work to illustrate the concepts explained in the remaining article, with Figure 1c representing a synthetic image.

From the user’s point of view, Level 1 (L1) images are easier to understand and to compare with maps or other images, whereas L0 images can be difficult to analyze and interpret (see Figure 1a). In this context, the geo-correction procedure can be viewed as creating an interface that makes images more easily readable by the analyst. However, geo-correction procedures require extensive computer resources and introduce spatial and spectral data errors in the very first stages of the image processing work flow.

Therefore, new image formats should take this schema into account by using the original spectral and spatial data contained within the same file. However, these new formats require the development of new image processing algorithms or the modification of existing ones for processing this type of file.

The consideration of positioning errors is very important when we compare images obtained from different platforms. This becomes especially obvious in quantitative image processing, where sub-pixel analysis is one of the most commonly used techniques. The level of accuracy achieved by this kind of analysis directly depends on the precision with which the pixel position and its spectral information is obtained by the sensor and preserved during subsequent pre-processing (from Level 0 to 3). This means that it is not possible to carry out an accurate sub-pixel analysis if the precise pixel position is not known. In addition, one and the same pixel of the geometrically corrected image may correspond to two different measurements performed by the sensor. Furthermore, one of the measurements may have been deleted during the re-sampling process, and thus, some of the information of the original data may have been lost, averaged out or, in the worst case scenario, even be false information, contributing to a loss of accuracy in the sub-pixel analysis. For all these reasons, the optimal data format must preserve the precision of both spatial and spectral information.

In this article, a new data structure for airborne hyperspectral images is presented. Such a structure attempts to minimize the impact of the aforementioned problems on the resulting images after processing them from Level 0 to 3.

The rest of the article is structured in the following way: Section 2 discusses the artefacts introduced by platform movement. Section 3 presents the proposed data structure, the Diffused Matrix Format (DMF), its two storage formats (disk and memory), and the application of basic image processing techniques using the DMF data structure. This is followed by an experiment that is performed to analyze the spatial pattern of the pixels using a sample image. In Section 4, a first experiment is carried out to determine the airborne image geo-correction error, and a second experiment includes a comparison between the different analyzed formats and the Diffused Matrix as related to the file size and the processing time. Finally, Section 5 presents the conclusions obtained in this study.

## 2. The Effects of Platform Instability on Airborne Hyperspectral Images

**Figure 2.**
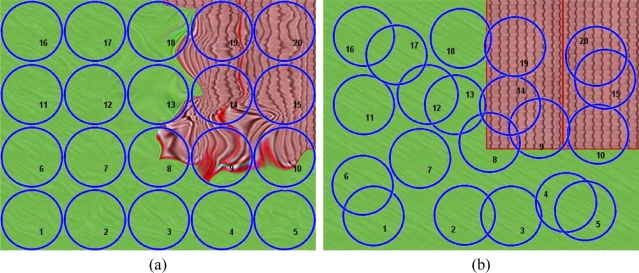
(a) L0 image showing the visual deformation of the scene. (b) Pixel's footprints over the ground truth image showing the real distribution of the measurements.

The random movement of an airborne platform carrying a hyperspectral sensor causes a displacement of the real position of the centroids of the acquired pixels. As a result, the pixels will not match any regular matrix pattern, as shown in Figure 2a. In this figure, the circles represent the pixel’s footprint. These measurements are placed according to their relative position in the L0 matrix without considering their real spatial location. Because of the pixel-spacing irregularity, L0 images always give the sensation of being distorted to the human eye.

Figure 2b shows the ground truth image that corresponds to the area where the real spatial locations of the pixels’ centroids were obtained from measurements by the GPS/INS system on board the platform. In Figures 2a,b, the footprint of the measurement’s target aim have been indexed for easier identification.

**Figure 3.**
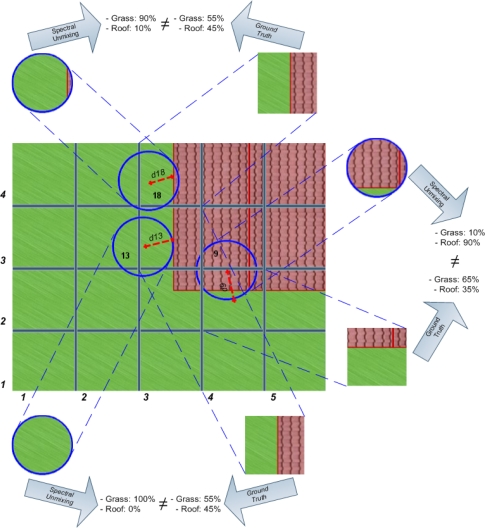
Erroneous unmixing results caused by geo-correction-induced displacements from the centroids’ values.

In order to show the effects that geo-correction procedures can have on measurement displacements, a grid has been superimposed on the image in Figure 3a that corresponds to the theoretical ground truth pixel distribution and reflects the real spatial location of surface features. The grid cells have been numbered consecutively starting from the lower left corner. In this case, measurement 13 was obtained over an area covered by grass only. This means that the spectral composition that can be derived from this measurement by spectral unmixing should be 100% grass and 0% of other constituents. However, the spectral composition in grid cell (3,3) of the ground truth image, where measurement 13 is being placed after geo-correction, is in reality 50% of grass and 45% of roof material and therefore, does not correspond to the assigned spectrum of 100% grass. This discrepancy is due to the fact that measurement 13 has been displaced by a distance of d13 from its real location, and with it its spectral information.

Obviously, this relocation problem affects not only a single pixel but most of the pixels in the geo-corrected image. An example is measurement 18, which has a composition of 90% of grass and 10% of roof and will replace (after geo-correction) the real composition of 55% of grass and 45% of roof at a grid cell position (4,3). Furthermore, measurement 9 has a surface composition of 10% of grass and 90% of roof that will substitute the real composition of 65% of grass and 35% of roof at grid cell (2,4).

**Figure 4.**
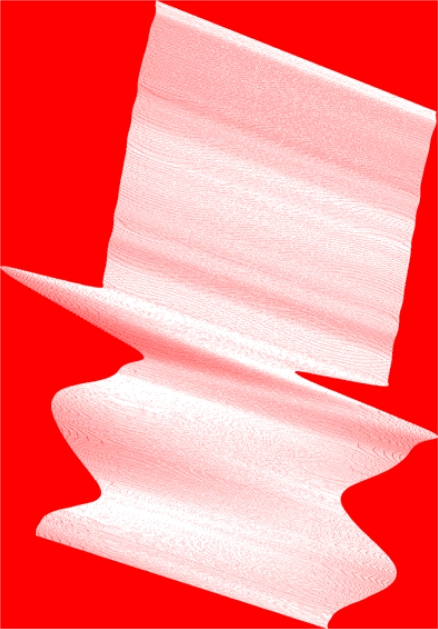
Representation of the Cuprite image showing the real location of the measurements acquired by the sensor (white) and the invalid pixels (red).

In order to analyze the randomness of the platform movement and the real position of the spectral measurements’ centroids, experiments were conducted using a hyperspectral image. The image is from the Cuprite area in Nevada, USA with a selected band presented in the L0 and geo-corrected format in Figure 1. This image was considered appropriate as it illustrates well the problem of platform movement. It was obtained by the AVIRIS NASA hyperspectral scanner [6], at an average height of 1590.82 m, and it is composed of 224 bands with a spatial grid of 1087 × 677 in its L0 state, and 1452 × 1010 after geo-correction. The aiming uncertainty error introduced by the GPS/INS system is 4 meters. This image is ideal for evaluating the efficiency of the proposed methodology, because, as it can be observed in Figure 4, the platform suffered random movements, resulting in a total of 48.475% of valid pixels (obtained from the provided GLT file), and a 51.525% of invalid pixels (re-assigned by proximity in the neighborhood and not-assigned). In this figure, the measurements' real location has been marked in white color, whereas the invalid pixels have been marked in red color.

The first experiment analyses the spatial distribution of the real measurements at various spatial positions within the image in order to obtain information if such a distribution presents a spatial regular behavior in the scene. The way to do this is by counting the number of the real pixel centroids (provided by the GPS/INS system) included in each latitude interval of the image (4 m). These numbers can be considered as density measurements per latitude, and should have some regular pattern according to the location of the pixels in the raster format (more measurements should be carried out near to the center of the pixels).

**Figure 5.**
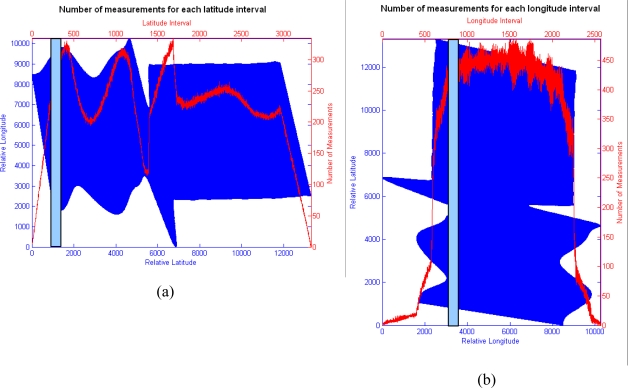
(a) Number of pixels for each latitude interval [4 × 4 m]. (b) Number of pixels for each longitude interval [4 × 4 m].

Figure 5 shows the number of points of the image that was measured by the sensor for each spatial interval of 4 meters (according to the uncertainty area). This interval has been represented as a blue bar in the corresponding figure. In Figure 5a, the density of points depends on the turbulences affecting each part of the image. The relative minimum counter value that appears near the 6,000 relative latitude corresponds to the zone of the image where the platform displacements have been severest. The two maximum values located near this minimum correspond to measurements displaced to nearby positions. However, the changes in density that appear in the right part of the image (from the relative latitude 7,000 to 12,000) cannot be justified by turbulences. The same procedure is carried out in Figure 5b, but using a 4-meter interval of longitude instead of latitude. The rapid changes in density that appear in the center of the image may correspond to noise. Inspection of the measurement distribution behavior in the image reveals no regular spatial pattern within the scene, meaning that the relative location of the pixel centroids of the points, where the valid measurements have been obtained, are totally random.

## 3. Application of a New Data Structure for Hyperspectral Image Processing

As mentioned in Section 1, currently one of the important problems when working with remote sensing images is the lack of a standard format used to store the information captured by the sensors. This means that suppliers deliver their products in their original formats as designed by the company or agency acquiring the data. Generally, a hyperspectral image is stored using four different files that can vary depending on the provider delivering the data: header file, IGM file, GLT file and data file [7]. However, these data file formats are not the only ones that exist, as there are many other alternatives such as GeoTIFF [8], ER Mapper [9], ERDAS IMAGINE [10]. From all formats, the internationally best known one is the Hierarchical Data Format (HDF) [11,12], which was designed by NASA and implemented in its Earth-Observing System (EOS) program.

### 3.1. The Proposed Storage Structure: the Diffused Matrix Format

It follows from the discussion in the previous sections that new standard data structures are needed to optimize the way in which Earth surface information is currently being stored. The main aspects that need to be improved in current formats are: spatial and spectral information preservation, minimization of the image file size, avoidance of duplicated information storage; and optimization of image processing in super-computer systems in order to facilitate the distribution of pieces of information.

**Figure 6.**
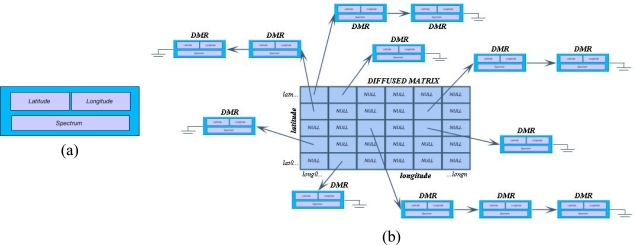
(a) A DMR structure. (b) Structure of the new proposed format, the Diffused Matrix Format (DMF).

In order to improve the performance of current formats according to the above listed requirements, we propose a new data structure called Diffused Matrix Format (DMF) [13,14]. This data structure is based on a measurement record (DMR), which stores information pertaining to each one of the actual measurements taken by the sensor. This information includes the UTM location of the pixel, and its spectrum (Figure 6a).

In this new data structure, the DMR records are placed in a grid called Diffused Matrix, according to their respective UTM location. This matrix is a regularly spaced grid that is indexed in N/S direction with respect to the latitude and longitude and in which the size of the elemental cell must be determined from the uncertainty of the pixel position due to the platform movements. A first approach consists of selecting a size in which each cell has at least one real value (DMR). Using this scheme, it is possible to place each one of the DMRs in a specific cell depending on its UTM location. This way, each measurement is automatically spatially placed in its respective cell, allowing faster image processing, without the necessity of running a geo-correction phase first.

One aspect that has to be taken into account is that spatially very close measurements may occur, meaning that more than one measurement may represent the same cell area (this situation occurs more often when the matrix resolution decreases, that is, when the cell size increases). In these cases, instead of averaging or substituting that particular cell, the DMR data structure will store all the corresponding real values. Therefore, as seen in Figure 6b, each matrix cell will be a pointer to a list of DMRs. This enables the user to always work with the real values, and it facilitates changing the image scale without losing information. This makes it possible to process the data even in extreme situations, for example when handling a maximum resolution image, which has a maximum of DMR in each cell, or a minimum matrix with a single cell that stores all the image data.

However, there is one case when the average spectrum of two measurements would need to be calculated in order to store only one measurement, and that is when both measurements are within the aiming uncertainty (*Δlat*, *Δlong*) of the GPS/INS positioning system. When this situation happens, calculating an average does not imply an information loss but a noise reduction.

Similarly, it is also possible to find cells with no values in the Diffused Matrix, because the sensor did not take any measurements in that specific spatial interval (NULL cells). These cells are excluded while processing the diffused matrix.

### 3.2. Storage of the Diffused Matrix

When storing the Diffused Matrix as a computer file, the structure itself is not being saved, rather all the DMR values are being stored one after another, resulting in a DMF file that is composed of a sequence of DMR records. This ensures that only valid measurements together with their geographical coordinates will be stored, while invalid values or averaged values will not, which is the case with the geo-corrected image. Therefore, the difference between the file size of the Diffused Matrix and that of the geo-corrected image will increase with increasing platform movements during data acquisition.

On the other hand, the use of the Diffused Matrix scheme comes with an extra storage cost that is caused by the inclusion of latitude and longitude values in the DMR registry, which are not being stored in conventional image files.

To load the file that contains the DMR, all that is needed is to read all the elements sequentially while the Diffused Matrix is being built, inserting each element in its corresponding cell according to its geographical coordinates.

### 3.3. DMF Image Processing

In order to analyze the performance of the DMF matrix, some basic techniques of conventional image processing were adapted to the DMF format:

- Thresholding Techniques: These operations consist of eliminating those pixels whose spectrum module does not surpass a predefined value. To execute this task, a conventional hyperspectral image must be first processed pixel by pixel before eliminating (by assigning a 0 value to its spectrum) those pixels whose Euclidean norm of the corresponding spectrum (||s||_2_, being *s* the spectrum) does not exceed the threshold value. In the proposed data structure, these operations are performed in a similar fashion, but with the added advantage that unless a measurement is assigned to a pixel, there is no need to use computing time in analyzing pixels without a measurement. On the other hand, there are cases where cells may have two or more values, making it necessary to follow the entire list of cells sequentially as shown in Figure 6.
- Convolution and Morphological Techniques: These operations consist of assigning a new value to each pixel after analyzing its neighbor pixels within a processing window called “processing kernel” that is moved over the entire image. Among the most popular techniques are, to name a few, the mean filter (convolution filters), and the dilation and erosion filters (morphological filters). The computation of these operations in the proposed data structure varies slightly from other data formats, because instead of using an *n* × *m* window size to search for neighboring pixels, a circle of radius *r* is used. In this case, all DMR elements that are located at a distance less than a given radius from the reference pixel are being considered.

## 4. Results

To test the accuracy of the different data structures, two further experiments were carried out. Experiment one focuses on the problems of geo-correcting airborne images as presented within this work. Experiment two presents accuracy improvement achieved when implementing the Diffused Matrix.

**Figure 7.**
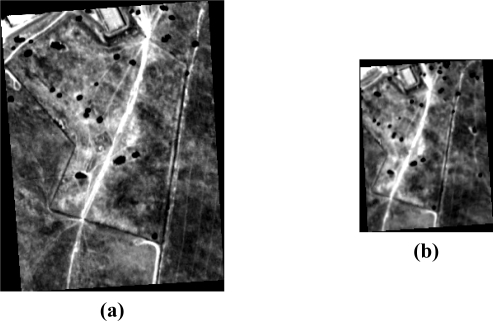
(a) Cáceres ***ROSIS_HR*** image. (b) Cáceres ***ROSIS_LR*** image.

For the first experiment, a study on geo-correction errors was developed [15]. To carry out this task, two geo-corrected images of the sensor ROSIS [16] were selected. The image area covers a small region near Cáceres (Spain) that includes *Quercus Ilex* trees, senescent vegetation (pasture), bare soil and shadow. The high resolution image (Figure 7a) was acquired in a low-altitude flight (ROSIS High Resolution image ***ROSIS_HR***), whereas the low resolution one was acquired in a high-altitude flight (ROSIS Low Resolution image ***ROSIS_LR***) (Figure 7b).

The aim of the experiment is to compare both images in order to detect the errors induced when geo-correction is performed.

No quantitative ground-truth map exists of this region. Therefore, it was necessary to build one using the information and data obtained from the ***ROSIS_HR*** image. The resulting ground-truth map should be an abundance map, where the pixels of each band would have information on the endmember's abundance values [17].

The ground-truth image was produced according to the following steps:

1. Determination of the endmembers of the ***ROSIS_HR*** image by using AMEE algorithm [18].
2. Classification of the high resolution image by using the Spectral Angle Mapper (SAM) algorithm and the endmembers obtained in step 1).
3. For every pixel i,j of the ROSIS_LR image the respective UTM coordinates x,y were obtained.
4. Search all the pixels of the ROSIS_HR image that are included in the footprint centered in x,y UTM coordinates.
5. The estimated abundance of the class_k for this pixel abundance_class_k_ROSIS_LR(i,j) is obtained by counting the number of high-resolution pixels classified as class_k in the foot print of the ROSIS_LR(i,j) pixel. The relative abundance is obtained by dividing abundance_class_k_ROSIS_LR(i,j) by the total number of pixels included in the foot print of the ROSIS_LR(i,j) pixel.

**Figure 8.**
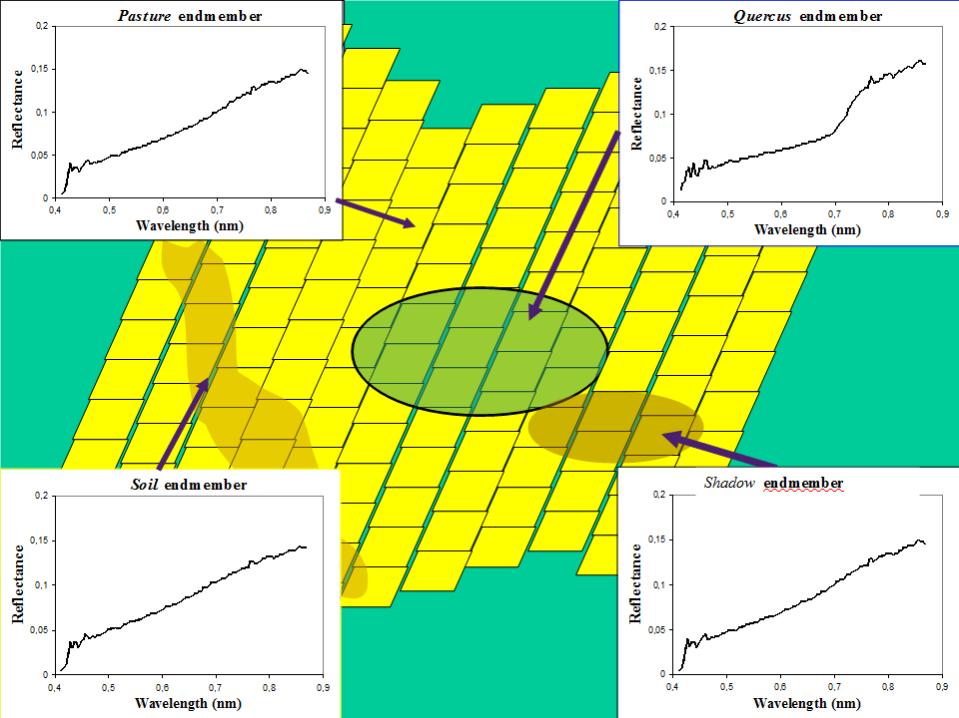
Spectral signatures of the endmembers and classification of the pixels included in the footprint of one pixel for the lowest resolution image.

Figure 8 presents the footprint (in green) of the ROSIS_LR(i,j) pixel superimposed on the pixels of the ROSIS_HR image (in yellow). The figure shows the endmembers for the ROSIS_LR image, and the results of the SAM classification.

**Figure 9.**
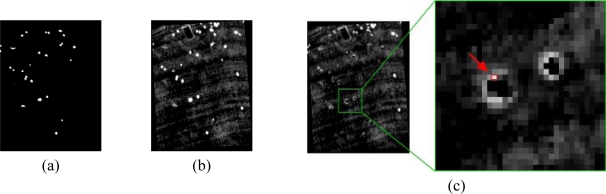
(a) *Quercus* ground truth abundance map for the ***ROSIS_LR*** image. (b) LSU *Quercus* abundance map for the ***ROSIS_LR*** image. (c) Difference between the abundance maps for the *Quercus* class.

Figure 9a shows the *Quercus* abundance ground truth map obtained by using the aforementioned algorithm for the ***ROSIS_LR*** image.

In order to obtain the abundance values from the ***ROSIS_LR*** image, an unmixing process has been carried out as follows:

- Determining the endmembers of the ***ROSIS_LR*** image by using the AMEE algorithm.
- Obtaining the abundance maps for the ***ROSIS_LR*** image by using the endmembers in the previous step and applying the LSU (Linear Spectral Unmixing) algorithm to the ***ROSIS_LR*** image.

Figure 9b shows the abundance map corresponding to the *Quercus* tree class obtained by using the LSU algorithm. In these figures, white pixels mean that the relative abundance of *Quercus* is almost 1. On the contrary, black pixels mean there is no *Quercus* abundance at all. The difference map between the abundances shown in Figure 9a and b has been calculated as an abundance difference map shown in Figure 9c, where the more white a pixel appears, the greater the differences in abundance values are.

As shown in Figure 9c, the highest abundance difference errors occur mostly along the edges of the *Quercus* trees that are present in the scene. By taking the pixel marked in red as an example, it can be seen that this individual pixels has been assigned a relative abundance of 100% *Quercus* tree. However, according to the ground-truth image, the real abundance of this element at that location is 0%. This means that a 100% error (white points) has been introduced. These false abundance values are due to pixel positioning errors introduced when geo-correcting the image because pixel spectra become displaced to their nearest neighboring pixel. The preservation of the original location by the DMF format avoids these artifacts that introduce spatial location errors that are not realistic for sub-pixel analysis.

A second experiment has been carried out to evaluate the efficiency of the proposed data structure. In this case, the Diffused Matrix structure is implemented using the image of Cuprite described in Section 2 as an example.

The implementation of such data structure was developed in C language, on Borland C++ Builder environment, and run on a Dual Core 2.10 computer, with 4 GB of RAM memory.

**Figure 10.**
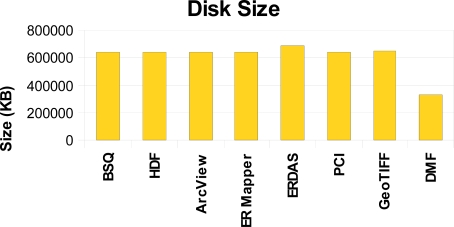
Comparison of the storage space of various image storage formats in relation to the Diffused Matrix (DMF).

In terms of analyzing the disk space requirements (Figure 10), the Diffused Matrix was built from the Cuprite image, using the L0 and IGM (coordinate location) information files, and stored as a vector of DMRs, as explained in Section 3.2. The size of the resulting file was compared to the size of the geo-corrected file of the Cuprite image by using several storage structures for hyperspectral images commonly used by current commercial providers in the market, as for example Band Sequential (BSQ), ArcView Raster, ER Mapper, ERDAS IMAGINE, PCI Geomatics (PCI), GeoTIFF and Hierarchical Data Format (HDF).

As shown in Figure 10, the disk space of the Diffused Matrix (DMF) is significantly less than the space required by the other common formats, because the DMF’s capability of storing only valid samples greatly reduces its disk space requirements. It therefore follows that the disk size difference, in relation to other formats, will increase the more movements (turbulences) the platform suffers.

**Figure 11.**
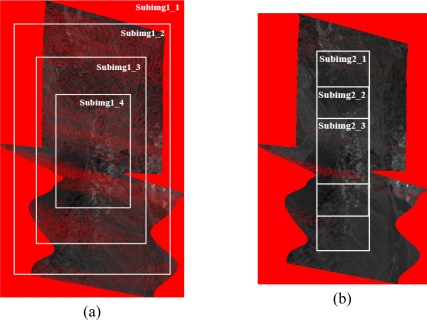
(a) First data set (SET 1). (b) Second data set (SET 2).

To compare the performance of the DMF with data sets which either include or exclude data that are located outside the original flight line (areas can be excluded by a mask), two data sets were obtained from the Cuprite image. The first set (Figure 11a) consists of constructing a diffused matrix with the same number of rows and columns as in the geo-corrected image. This set was gradually reduced into sub-images that show high densities of measurements. The second set (Figure 11b) consists of constructing a diffused matrix with the same number of rows and columns as in the L0 image. As seen in Section 2, this image is smaller than the geo-corrected one, so the resulting diffused matrix will also be smaller than the one in the first set and will contain longer DMR lists than the previous set (higher density of measurements). As in the previous case, this region was gradually reduced to various sub-images, but with similar high density measurement areas.

Both sets, together with their analogous geo-corrected versions, have been subjected to two very different kinds of processing methodologies. The first one is a thresholding technique where a euclidean norm threshold value of 47000 was used. In this way, for each pixel (i,j) of the image, whose spectrum can be represented as s_(i,j)_, the Euclidean norm ||s_(i,j)|_||_2_ was calculated. So, those pixels whose euclidean norm was lower than 47000 were deleted (the spectra were set to 0).

The second one consists of an erosion filter. First of all, this kind of filtering was applied to the geo-corrected image using a square kernel of 3 × 3. In order to carry out impartial comparisons, a square kernel of 3 × 3 should also be applied to the first and the second set, where the image is represented in Diffused Matrix formats. But, as commented in Section 3, the thresholding filtering using DMF images requires the use of a circular kernel with radius *r*. Because the matrices in the first and the second set had different sizes (regarding the number of cells in columns and rows), and therefore their cell size was different, a radius of respectively 35 m and a 25 m was used for them. In this way, it is not necessary to analyze measurements farther than a cell kernel of 3 × 3 for each DMF image.

**Figure 12.**
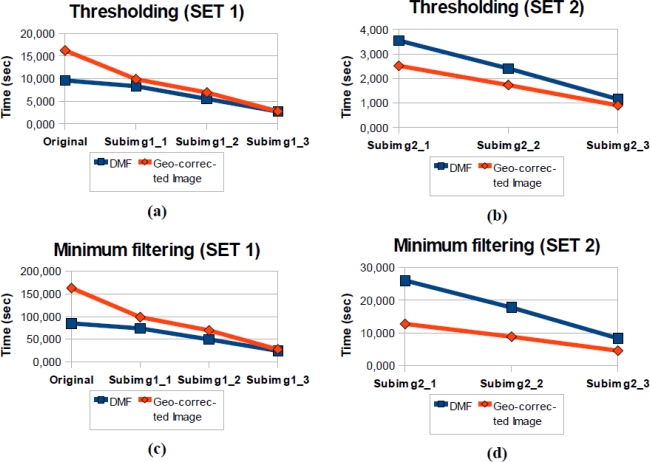
(a) Thresholding results for the first data set. (b) Thresholding results for the second data set. (c) Erosion filtering results for the first data set. (d) Erosion filtering results for the second data set.

Figure 12 shows the processing time tables for computing the mentioned operations on both data types.

The results show that the processing times for the first set of images (Figure 12a) are less than the results for the same operations on the second set (Figure 12b), which consists of the hyperspectral geo-corrected image. The reason for this performance difference is that there is a high amount of non-assigned pixels that are not analyzed in the Diffused Matrix (the first set), but they are analyzed in the geo-corrected image, which is why this feature involves a considerable saving of processing time.

However, there are differences observed in the second set of images (Figure 12c and Figure 12d), although the matrix has a smaller size and the number of cells to be analyzed, has decreased. This is due to a larger number of DMRs that exist in each cell and the reason why the time elapsed in processing the lists has increased.

## 5. Conclusions

This article presents a study of the spatial errors produced when acquiring the data from airborne sensors due to the turbulences the airplane is subjected to, and the subsequent pixel displacements that occur when geo-correcting the resultant image.

To test this phenomenon, three different hyperspectral images have been used and several experiments have been carried out.

The first image was acquired by a scanner sensor whereas the second and the third images were acquired by a push broom sensor. They were captured from different acquisition platforms at different altitudes, and all of them were affected by the movement of their platforms caused by turbulences.

Regarding the experiments, the first one, presented in Section 2, details that it is possible to deduce that no regular spatial pattern appears in the scene and most of the real measurements are obtained at a random distance from the center of the pixels of the image grid. In the second set of experiments outlined in Section 4, the first experiment shows the importance of these errors on real quantitative maps. These facts confirm that the storage of the real spatial location of the measurements is fundamental for accurate quantitative remote sensing analysis. Therefore, it is not possible to obtain an accurate map with quantitative sub-pixel information by using raster image format because of the spatial errors induced by these data structure.

To solve the above outlined problems when acquiring the data from airborne sensors, a new data structure that enables improved data storage of airborne hyperspectral images has been designed. This structure consists of a special matrix (Diffused Matrix) that only stores the real values as measured by the sensor, including the UTM coordinates of each measurement, as a DMR record. This design provides enormous advantages, since, conversely to conventional formats, invalid or averaged pixels are discarded, which is why the margin of processing error of this kind of images is considerably reduced.

The new file format consists of a sequential file that stores all the DMR records. The fact that no invalid or averaged pixels, as they often occur in hyperspectral geo-corrected images, are being stored allows a significant reduction of the disk size, which normally becomes larger as the platform is subjected to increased movements during data acquisition.

The last experiment, outlined in Section 4, focuses on the performance analysis of the Diffused Matrix. Concerning the application of basic image processing techniques, the proposed data structure shows overall good performance especially when the density of measurements contained in the DMF cells is low and platforms are subjected to increased movements. In DMF image areas where measurement densities are high, the DMR lists need to be processed sequentially, in which case the cost of the execution time is similar to the cost of the conventional processing.

Future lines of investigation will focus on:

1. The solution of this problem using parallel processing techniques in heterogeneous computer clusters and exploring non sequential possibilities for the DMF data structure.
2. The application of the method to the in-field separation of spectral bands.
3. To address cases of airborne hyperspectral sensors (where other non-geometrical problems, such as spectral smile or spectral broadening effects appears).
